# Thermosensitive Hydrogel-Functionalized Mesoporous Silica Nanoparticles for Parenteral Application of Chemotherapeutics

**DOI:** 10.3390/gels9090769

**Published:** 2023-09-21

**Authors:** Christina Voycheva, Marta Slavkova, Teodora Popova, Diana Tzankova, Denitsa Stefanova, Virginia Tzankova, Ivelina Ivanova, Stanislav Tzankov, Ivanka Spassova, Daniela Kovacheva, Borislav Tzankov

**Affiliations:** 1 Department Pharmaceutical Technology and Biopharmacy, Faculty of Pharmacy, Medical University—Sofia, 1000 Sofia, Bulgaria; hvoycheva@pharmfac.mu-sofia.bg (C.V.); tpopova@pharmfac.mu-sofia.bg (T.P.); btzankov@pharmfac.mu-sofia.bg (B.T.); 2 Department of Pharmaceutical Chemistry, Faculty of Pharmacy, Medical University—Sofia, 1000 Sofia, Bulgaria; d.tsankova@pharmfac.mu-sofia.bg; 3 Department of Pharmacology, Pharmacotherapy and Toxicology, Faculty of Pharmacy, Medical University—Sofia, 1000 Sofia, Bulgaria; denitsa.stefanova@pharmfac.mu-sofia.bg (D.S.); vtzankova@pharmfac.mu-sofia.bg (V.T.); 4 Faculty of Pharmacy, Medical University—Pleven, 5800 Pleven, Bulgaria; ivelinavasileva23@gmail.com (I.I.); stanislav_tzankov@abv.bg (S.T.); 5 Institute of General and Inorganic Chemistry, Bulgarian Academy of Sciences, 1113 Sofia, Bulgaria; ispasova@svr.igic.bas.bg (I.S.); didka@svr.igic.bas.bg (D.K.)

**Keywords:** hydrogel gatekeeper, mesoporous silica nanoparticles, stimuli-sensitive delivery, chemotherapy, doxorubicin

## Abstract

Hydrogels can offer many opportunities for drug delivery strategies. They can be used on their own, or their benefits can be further exploited in combination with other nanocarriers. Intelligent hydrogels that react to changes in the surrounding environment can be utilized as gatekeepers and provide sustained on-demand drug release. In this study, a hybrid nanosystem for temperature- and pH-sensitive delivery was prepared from MCM-41 nanoparticles grafted with a newly synthesized thermosensitive hydrogel (MCM-41/AA-g-PnVCL). The initial particles were chemically modified by the attachment of carboxyl groups. Later, they were grafted with agar (AA) and vinylcaprolactam (VCL) by free radical polymerization. Doxorubicin was applied as a model hydrophilic chemotherapeutic drug. The successful formulation was confirmed by FT-IR and TGA. Transmission electron microscopy and dynamic light scattering analysis showed small particles with negative zeta potential. Their release behaviour was investigated in vitro in media with different pH and at different temperatures. Under tumour simulating conditions (40 °C and pH 4.0), doxorubicin was almost completely released within 72 h. The biocompatibility of the proposed nanoparticles was demonstrated by in vitro haemolysis assay. These results suggest the possible parenteral application of the newly prepared hydrogel-functionalized mesoporous silica nanoparticles for temperature-sensitive and pH-triggered drug delivery at the tumour site.

## 1. Introduction

Conventional chemotherapy is a mainstay of treatment for various cancer types [1]. It relies on the application of a variety of drug agents that can kill the cancerous cells. Unfortunately, their efficacy is usually hampered due to their inherent side effects on healthy tissues [2]. Another limitation is the need for frequent applications, which imposes an immense burden on the patients’ quality of life. Therefore, significant trust and scientific effort has been put into the nanotechnological approach to overcome all the hurdles associated with chemotherapy. Various types of nanocarriers have been prepared and loaded with different chemotherapeutics, aiming to achieve improved antitumour efficacy, prolonged and sustained release, and to lessen the adverse events [3,4,5,6,7,8].

Hydrogels are a class of highly studied drug carriers with good biocompatibility and the possibility to be tuned for desirable properties and different routes of administration [9]. Hydrogels exhibit a three-dimensional structure of interconnected and crosslinked polymers that can absorb extensive amounts of water and yet do not dissolve in water [10]. Recently, attention has been directed towards intelligent hydrogels. They possess stimulus-sensitive behaviour triggered by alterations in the environmental conditions, such as temperature, pH, redox potential, light and others [11,12]. Amongst them, pH- and thermo-sensitivity have been exploited greatly in chemotherapy. This is mainly due to the fact that tumour cells are characterized by a specific microenvironment with an acidic medium and hyperthermia because of their intensive metabolism and vascularization [13,14]. Thus, a hydrogel with appropriate modifications can undergo reversible sol-gel transition at a specific desired temperature and release the loaded drug [15]. Depending on the temperature that causes the sol-gel transition, the hydrogels can have either a lower critical solution temperature (LCST) for the hydrophilic to hydrophobic transition or an upper critical solution temperature (UCST) for the opposite behaviour. In pharmaceutical applications, substances with LCST are widely used, such as poly(N-isopropyl acrylamide) (PNIPAM), poly(N-vinylcaprolactam) (PVCL), poly(N-vinylalkylamide), poly(2-hydroxyethylmethacrylate) (PHEMA), pluronic, chitosan, hyaluronic acid, etc. [16]. The specific temperature causing the on-demand drug release can be finely tuned by chemical or physical modifications of the parent polymer [15]. Nevertheless, there are some challenges in the application of hydrogels in chemotherapy, such as their lower mechanical strength and troublesome sustained release, especially in the case of hydrophilic drugs [17,18]. 

Developing composite systems for drug delivery has been shown to surmount the limitations of either one of their components on its own [19]. These are hybrid nanocarriers that combine the properties of different carriers, i.e., hydrogel and inorganic nanoparticles [20]. Different examples of their combinations can be found in the literature [21,22,23]. Thermosensitive hydrogels have been used by other researchers as gatekeepers to achieve sustained and specific drug delivery [16,24]. Some very suitable inorganic nanocarriers to be functionalized with appropriate hydrogel gatekeeper are mesoporous silica nanoparticles (MSNs) [19,25]. MSNs are characterized by a high specific surface area, an adjustable pore size, high hydrothermal stability, a large pore volume, biocompatibility and very low toxicity [19,26,27,28,29]. Moreover, their internal and external surface is highly covered with silanol groups, which can easily undergo functionalization [29]. The introduction of new chemical modalities (such as carboxylic groups) could be further utilized as a site for grafting with polymers, i.e., hydrogels [30].

Natural polymers are very attractive for modification because of their nontoxicity and biodegradability. Marine polysaccharides are extracted from renewable sources and are cost-effective [31]. Agar is a polysaccharide derived from algae which can form a strong biocompatible hydrogel. As an excipient, it is widely used in pharmaceutical practice in the role of a binder, filler, thickening agent, gelling agent, etc. [31,32]. Agar on its own does not possess thermosensitive properties, but can easily be modified by suitable grafting and result in a thermoresponsive hydrogel [33]. Therefore, agar was selected as a backbone for the gatekeeper modification of MSNs in the current study. 

The present research aimed to obtain hydrogel-functionalized mesoporous silica nanoparticles for thermosensitive drug delivery. Doxorubicin was selected as a model drug due to its hydrophilic nature and versatile application in many forms of solid tumours and leukaemia [34]. It was hypothesized that the hybrid nanoparticles would be a suitable candidate for parenteral chemotherapy. The biocompatibility of the proposed nanocarrier was evaluated through its haemolytic potential.

## 2. Results and Discussion

The novel hydrogel AA-g-PnVCL was synthesized using a free-radical-initiated polymerization in a nitrogen atmosphere, as the amount of monomer and the concentration of the radical initiator were chosen on the basis of previous work. The rationale for the chosen ratios between the components of the hydrogel lies in the LCST (40 °C) of the obtained polymer together with its sufficient grafting efficiency (62.4%) [33]. The preparation of the hydrogel was based on the grafting-from strategy, where the polymerization occurs directly at the MSN’s surface [35,36]. 

The LCST was chosen to be 40 °C, as solid tumours, in comparison with healthy tissue, are characterized by elevated temperatures (around 40–43 °C) [37,38]. In addition, this temperature is considered to be mild hyperthermia that can be applied locally alongside the chemotherapy and further improve the outcome with minimal discomfort to the patient [38].

### 2.1. Synthesis of Hydrogel-Functionalized Nanoparticles MCM-41/AA-g-PnVCL

**Figure 1:**
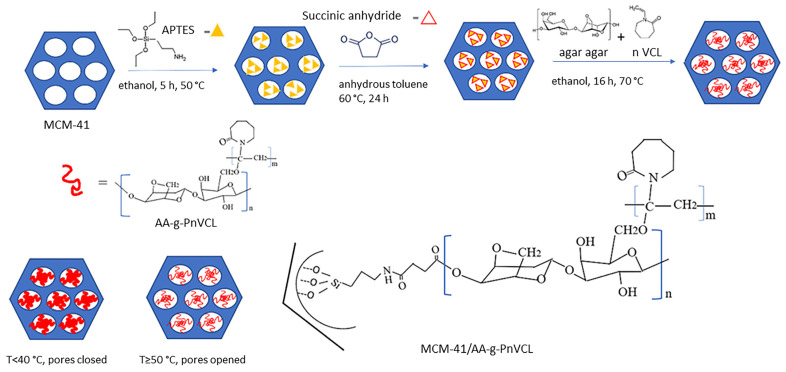
Synthesis of the hydrogel-functionalized silica nanoparticles.

Here, a two-step-controlled grafting method is proposed. First, functionalization (carboxylation) of the mesopores and the surface of MCM-41 was performed, followed by free radical polymerization of AA and nVCL in the presence of carboxyl modified MCM-41, using 2,2′-azobis(2,4-dimethylvaleronitrile) (AMVN) as a free radical initiator (Figure 1). The carboxyl modification of MCM-41 was carried out to provide reactive sites for attaching AA-g-PnVCL. The hydrogel was synthesized in the presence of carboxyl-modified MCM-41.

### 2.2. Characterization of the AA-g-PnVCL and MCM-41/AA-g-PnVCL

#### 2.2.1. FT-IR

**Figure 2:**
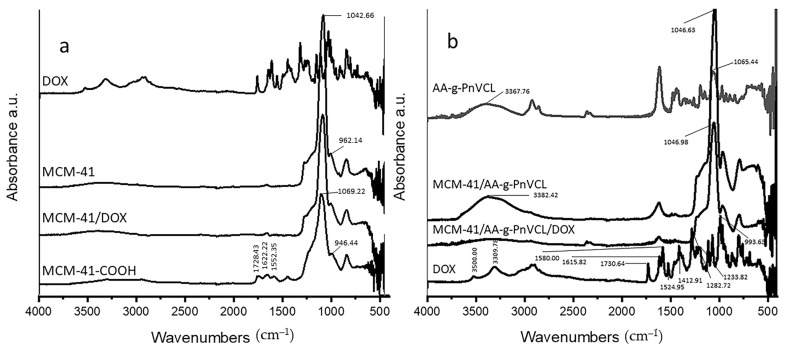
FT-IR spectra of (**a**) Dox, the parent MCM-41, Dox-loaded MCM-41 and carboxyl-modified MCM-41, and (**b**) Dox, AA-g-PnVCL, MCM-41/AA-g-PnVCL and loaded hydrogel-functionalized nanoparticles.

The FT-IR spectra of all samples are presented in Figure 2. The parent silica MCM-41 materials were characterized by an intensive band at 1042 cm^−1^, which was due to the asymmetric stretching vibration of the silica structure (Si–O–Si); the band at 962 cm^–1^ corresponded to the stretching vibrations of the surface Si–O groups [39,40]. After Dox loading onto MCM-41, no shifting of the silanol group bands was observed. It can be assumed that the Dox was physically entrapped in the nanocarriers. The significant shifting of the silanol group bands to 1069 cm^−1^ and 946 cm^−1^, and the appearance of N-H bending at 1552 cm^−1^, as well as the stretching vibration of the amide group at 1622 cm^−1^ (C=O) and the carboxylic groups (C=O) at 1728 cm^−1^ showed the successful attachment of COOH to the MCM-41 [41]. The reaction between APTES and succinic anhydride led to the formation of an amide bond, which was represented in the spectrum of MCM-41-COOH. A new peak also appeared from the attached COOH groups from the succinic anhydride.

The shifting of the characteristic band of hydroxyl groups to 3382 cm^−1^ and the disappearance of the CH_2_OH band visible at 1065 cm^−1^ in the spectrum of AA-g-PnVCL were evidence of the successful grafting of AA-g-PnVCL on MCM-41-COOH. Doxorubicin showed stretching vibrations of O-H and N-H bonds in the range of 3300–3550 cm^−1^. The peak at 1730 cm^−1^ correlated with COOH groups, while those at 1615 cm^−1^ and 1580 cm^−1^ correlated with the ring (phenol), 1524 cm^−1^ correlated with the aromatic ring (C=C), 1412 cm^−1^ correlated with (N–H), 1282 cm^−1^ correlated with (C–C), 1233 cm^−1^ correlated with (C–N) and 993 cm^−1^ correlated with (C–OH), and were confirmed with literature data [42].

The spectrum of Dox loaded on the hybrid MCM-41/AA-g-PnVCL nanoparticles showed only a lowering of the intensities, but no significant shifting in the characteristic peaks. This fact suggests that doxorubicin was physically included in the MCM-41/AA-g-PnVCL particles.

#### 2.2.2. TEM and SEM Analysis

**Figure 3:**
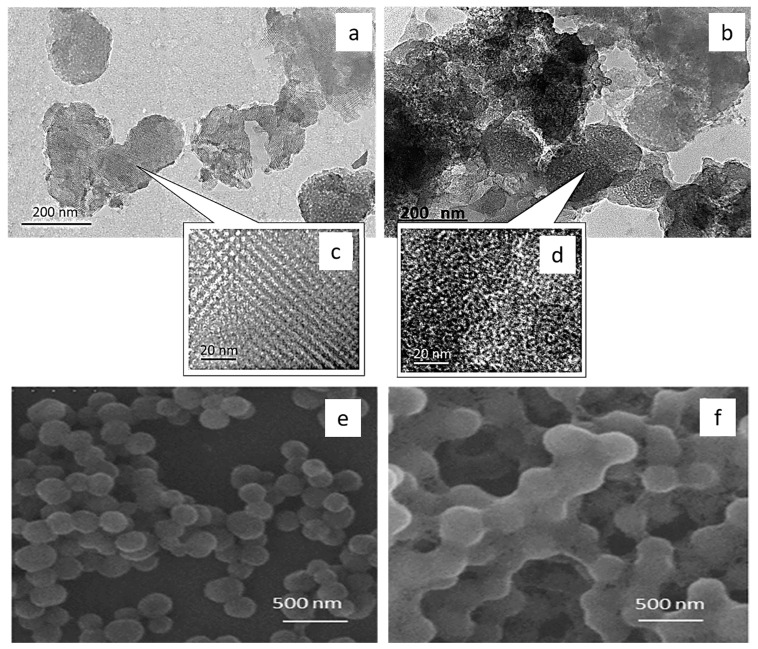
Transmission electron microscopy (**a**–**d**) and scanning electron microscopy (**e**,**f**) images of MCM-41 (**a**,**c**,**e**) and MCM-41/AA-g-PnVCL (**b**,**d**,**f**).

TEM studies were performed for analysing the surface structure of MCM-41 and MCM-41/AA-g-PnVCL (Figure 3a–d). As seen in the figure, MCM–41 had the expected porous structure and spherical shape (Figure 3a,c). In accordance with the XRD data, the images of MCM-41/AA-g-PnVCL particles (Figure 3b,d) also showed some differences in the surface structure. This fact could be explained by pore blockage caused by the hydrogel modification of the particles. However, the hydrogel on the surface of silica particles did not significantly influence the morphology of MCM-41.

SEM images (Figure 3e,f) showed the spherical shape of the particles and sizes between 200 and 300 nm. The differences in the particles’ size could be explained by the hydrogel crown formed around the MCM-41’s surface due to grafting with AA-g-PnVCL (Figure 3f).

#### 2.2.3. TGA

**Figure 4:**
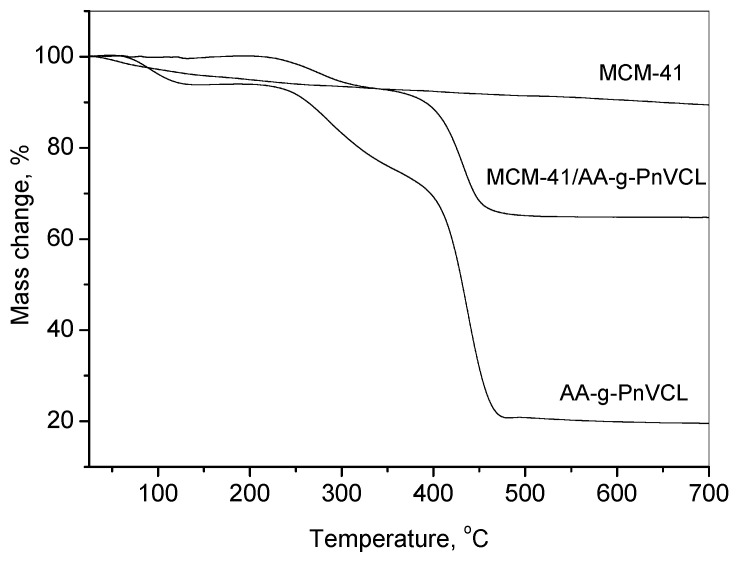
TG-curves of MCM-41 and MCM-41/AA-g-PnVCL and AA-g-PnVCL.

The attachment of AA-g-PnVCL on the MCM-41 particles could be evidenced by the thermogravimetric analyses shown in Figure 4. It presents the TG curves of MCM-41, the AA-g-PnVCL hydrogel and the hybrid system MCM-41/AA-g-PnVCL in the temperature range of 25–700 °C. Three main thermal steps were registered for the hydrogel.

The first temperature weight loss ranged from 60 °C to about 120 °C, where the polymer lost about 8% of its mass due to dehydration. The second step was from 205 °C to 340 °C due to the depolymerization of the agar-agar, with a loss of about 25% of its mass. The third decomposition step occurred from about 340 °C to nearly 475 °C, where 35% of the material’s mass was lost, probably due to the degradation of nVCL units with a full mass loss of nearly 80%. One could observe that the pure MCM-41 particles had a gradual and slight weight loss across the whole temperature range (10%), while the thermal decomposition stages of MCM-41/AA-g-PnVCL appeared at the same stages and temperatures as AA-g-PnVCL, and the difference between the mass loss of MCM-41 and MCM-41/AA-g-PnVCL was about 25%. The residue of pure AA-g-PnVCL was about 20%. The amount of attached polymer was calculated on the basis of the mass losses of the individual compounds and the MCM-41/AA-g-PnVCL sample. Since the residue of MCM at 700 °C was 90% and that of the AA-g-PnVCL was 20%, from the value obtained for the residue of the MCM-41/AA-g-PnVCL sample (65%), the quantity of the polymer bonded was found to be 36% of the initial mass. Hence, there was a successful attachment of the AA-g-PnVCL on the MCM-41 particles.

#### 2.2.4. XRD

**Figure 5:**
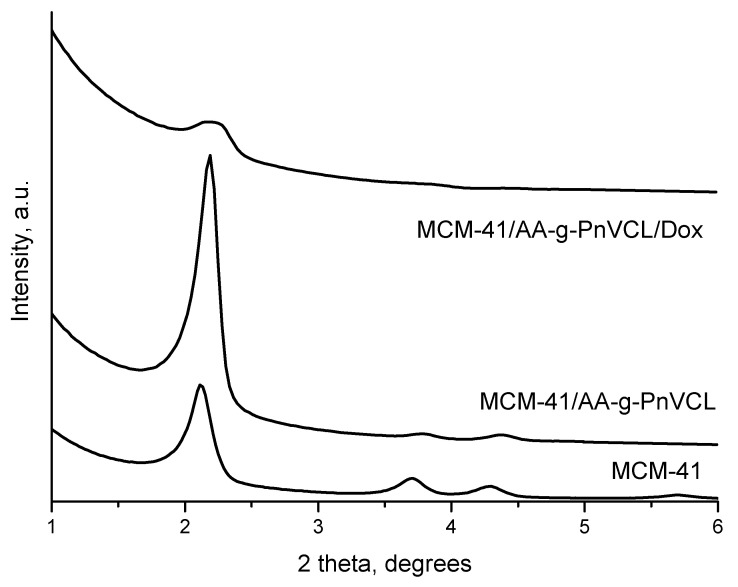
The small-angle part of the XRD patterns of MCM-41, MCM-41/AA-g-PnVCL and MCM-41/AA-g-PnVCL/Dox.

The small-angle parts of the XRD patterns of MCM-41 and MCM-41/AA-g-PnVCL and MCM-41/AA-g-PnVCL/Dox are presented in Figure 5. The X-ray powder diffraction pattern of the MCM-41 material exhibited four pronounced peaks, namely the most intense at (100) and the well-split (110) and (200), along with (210) reflections as an indication of the hexagonal pore structure with a high degree of ordering. The unit cell’s parameter was 46.79(5) Å. The pattern of the MCM-41/AA-g-PnVCL showed a slight shift of the peaks towards higher degrees of 2 theta and peak asymmetry, which could be attributed to the changes in the pore walls’ thickness due to the attachment of the AA-g-PnVCL on MCM-41. The pattern of the MCM-41/AA-g-PnVCL/Dox showed only one peak with low intensity, indicative of the filling of the mesopores with Dox, which was a confirmation of successful loading of the drug.

#### 2.2.5. Temperature-Sensitive Behaviour

**Figure 6:**
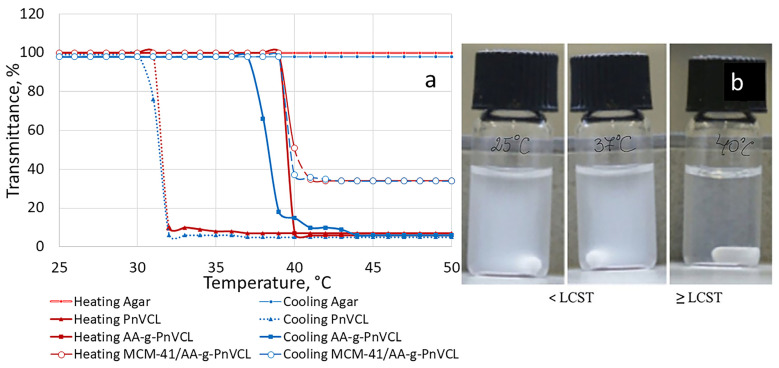
(**a**) UV transmittance at 500 nm as a function of the temperature of agar, PnVCL, AA-g-PnVCL and MCM-41-AA-g in a diluted aqueous dispersion (4 mg/mL). (**b**) Macroscopic photos of the corresponding hydrogel-functionalized nanoparticles’ dispersion at various temperatures.

AA has no temperature sensitivity, while the LCST of PnVCL is 33 °C, as can be seen in Figure 6a. Nevertheless, this temperature is lower than the normal body temperature. Therefore, each of the components on their own did not provide sufficient thermoresponsive properties for modified drug release purposes. By grafting nVCL units on the AA hydrophilic backbone, the desirable thermosensitivity was achieved. Many researchers have discussed the application of nVCL in the development of thermosensitive drug delivery systems, e.g., microgels, hydrogels, nanogels and others [43]. It has been established that the change in the ratio of the hydrophilic polymer and the hydrophobic monomer gives different temperature behaviour of the polymers. The synthesized polymer AA-g-PnVCL (Figure 6a) at a predetermined ratio of the components had the characteristics necessary for the subsequent work with doxorubicin loading and achieving smart release at a temperature of 40 °C [33]. Other research groups have studied the grafting of different macromonomers with nVCL and their effect on the LCST of the resulting graft polymer. Chitosan was successfully used in combination with different ratios of PnVCL to produce a thermosensitive polymer with an LCST above normal body temperature for the delivery of 5-fluorouracil [44] or cisplatin [45]. Furthermore, the study of Banihasem et al. [45] showed that a coating of gold nanoparticles on the grafted chitosan–PnVCL polymer did not significantly affect the thermosensitive behaviour of the nanocarrier and allowed modified release of the chemotherapeutic.

Thermoresponsive polymers have a homogenous aqueous solution below the LCST and undergo a heterogeneous disperse system change above it. They prevent the release of the drug before the LCST is reached and stimulated the delivery of the active substance above LCST in larger quantities when the utility is the largest. The sharp changes from a swollen to a collapsed state are an opportunity to use this hydrogel as a gatekeeper and release the anticancer drug in a smart temperature-sensitive manner.

The grafting of MCM-41 nanoparticles with the synthesized thermosensitive hydrogel with a LCST value higher than usual body temperature could keep the API in the mesopores, preventing or drastically slowing its release below a certain temperature and hastening it if the local temperature is above the polymer’s LCST. The thermoresponsive behaviour of hybrid nanoparticles is evident in Figure 6a,b. At 40 °C, the turbidity decreased sharply for both the hydrogel and the hydrogel-modified nanoparticles (Figure 6a). This observation gives us the reason to assume that MCM-41 does not significantly change the behaviour of AA-g-PnVCL after grafting. Similar results have been achieved by other authors. Karesoja et al. [46] investigated the thermosensitivity of mesoporous silica nanoparticles directly grafted with VCL and further modified with polyethylene oxide. The obtained nanoparticles possessed an LCST in the range of 35–37 °C. The closing of the pores of the mesoporous silica nanoparticles was also achieved by Sing et al. with the help of NIPAm-PEG [47]. The obtained system showed sustained of release Dox at temperatures below the LCST of the polymer and triggered its release above the LCST. The drug’s release can be provoked by applying exterior heat to the field with the tumour tissue or by using the natural difference between healthy and tumour tissues, known as local tumour hyperaemia [37,38,48].

#### 2.2.6. Dynamic Light Scattering (DLS)

Pristine MCM-41 nanoparticles are characterized by a small particle size and negative zeta potential, which is in accordance with the literature data [49,50]. The negative charge is due to the presence of silanol groups on their surface, which undergo deprotonation in an aqueous medium. Measurements performed by DLS analysis showed an increased particle size and polydispersity index after grafting of the polymer on the surface of the MCM-41 particles and a negative zeta potential above 20 mV (Table 1). The engagement of surface silanol groups in the graft with the thermosensitive polymer led to a decrease in the absolute value of zeta potential. Similar results after grafting were reported by other publications [49]. The value of the polydispersity index (PDI) showed the stability of the parent particles and the size distribution within narrow limits. The polymer graft predominantly affected the surface of the carboxyl-modified MCM-41, as can be seen from the TEM images as well (Figure 3b). Therefore, an increase in particle was observed, as expected due to the attached polymer chains. The increase in the PDI after preparation could be explained by the different number of grafted polymer molecules on each MCM-41-COOH particle. The decrease in the absolute zeta potential may also be a reason for the aggregation of particles and increased polydispersity. After Dox loading, the PDI and size of the particles remained in the same range as the ones of the Dox-free hybrid nanoparticles. These results suggested that Dox does not affect the size of the prepared hybrid nanoparticles, and no significant change was observed in their particle size distribution. The decrease in the absolute value of the zeta potential could be due to an electrostatic interaction between the nanocarrier and Dox. It is well known that Dox is a cationic drug (pKa = 8.3) which is positively charged in physiological conditions due to the presence of amine groups [51,52]. The results for the average size are in agreement with the TEM analysis.

#### 2.2.7. Dox Loading Efficiency

API loading following post-surface grafting was chosen in the present study. According to the literature data, it has been suggested that during the numerous steps of grafting and extraction, Dox can be lost from the nanocarriers [53]. The loading of Dox onto unmodified MCM-41 was found to be 49%, while the loading efficiency on MCM-41/AA-g-PnVCL was calculated to be 59%. It was expected that the active substance was physically encapsulated in the grafted MCM-41 particles, according to the results of IR spectroscopy. The higher loading capacity of the hybrid particles was probably due to the formation of internal free volumes from the chains of AA-g-PnVCL attached to the surface of MCM-41.

#### 2.2.8. Drug Release

**Figure 7:**
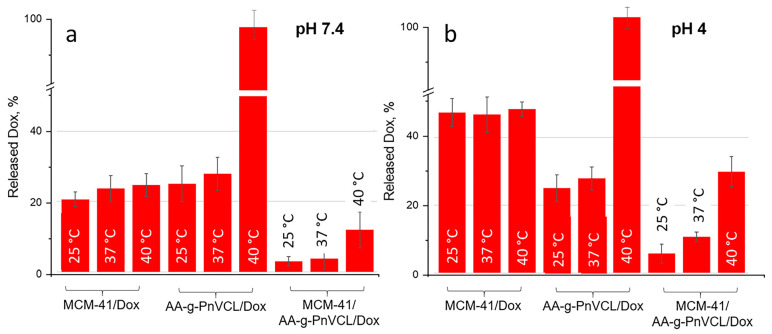
Amount of doxorubicin released (%) in the sixth hour during an in vitro dissolution test at pH 7.4 (**a**) and pH 4.0 (**b**) at different temperatures (25 °C; 37 °C; 40 °C); mean ± SD; n = 3.

**Figure 8:**
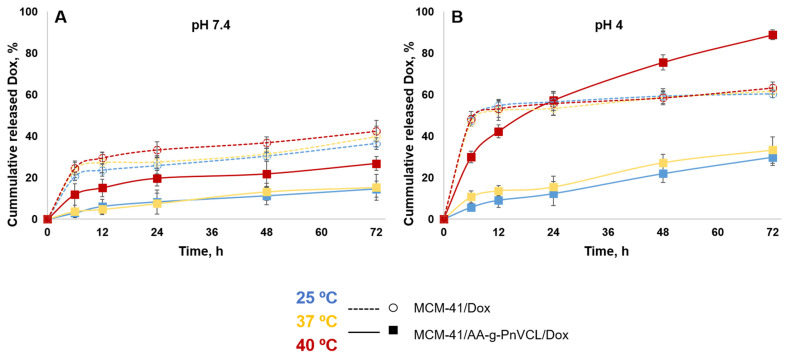
Release profiles of doxorubicin from MCM-41/AA-g-PnVCL and MCM-41 nanoparticles in a buffer medium with a pH of 7.4 (**A**) and a pH of 4 (**B**) at different temperatures (25 °C; 37 °C; 40 °C); mean ± SD; n = 3.

The expected temperature- and pH-dependent release of Dox was monitored by performing an in vitro dissolution test in a buffer medium (pH 7.4 and 4.0) at 25 °C, 37 °C and 40 °C, corresponding to room temperature, normal body temperature and tumour hyperaemia. The results are presented in Figure 7 and Figure 8.

The release of doxorubicin from unmodified MCM-41/Dox at pH 7.4 was slow (20.6 ± 2.1%) within the first 6 h of the study (Figure 7a). At pH 7.4, the Dox molecules were partially protonated, as expected on the basis of their pKa = 8.22 [52,53]. Therefore, at this pH, Dox was positively charged while the MSN carrier’s silanol groups were characterized by a negative charge. This led to electrostatic attraction between the carrier and the API, and the drug was retained. These results are in accordance with other studies [48,49,50,51]. In the 72nd hour at pH 7.4 (Figure 8A), regardless of the temperature, about 40% was released from the nonmodified particles (36.5 ± 2.8% at 25 °C; 39.8 ± 4.9% at 37 °C; 42.4 ± 5.2% at 40 °C). This suggests incomplete but still sufficient release of Dox at normal physiological conditions, which could cause toxic systemic effects.

At the same time, the thermosensitive AA-g-PnVCL/Dox showed complete release of Dox only at 40 °C (Figure 7a,b), regardless of the pH of the medium (97 ± 3.4% at pH 7.4 and 96.8 ± 3.8% at pH 4). The results obtained from the in vitro dissolution test for the hydrogel-functionalized nanoparticles at 25 °C and 37 °C showed a delayed release in the initial phase, as well as a slow release over time. Less than 5% (2.9 ± 1.3% and 3.7 ± 3.0% at 25 °C and 37 °C, respectively) of the active substance was released in the first 6 h (Figure 7a), followed by about 14.5 ± 3.9% and 15.3 ± 6.3%, respectively, at 25 °C and 37 °C being released by the 72nd hour (Figure 8A). This delay in the release was expected due to the swollen state of the hydrogel at room temperature and normal body temperature. This suggests the possibility for temperature-sensitive drug delivery at the tumour site, as shown in the literature [33]. The release of Dox from the hybrid nanoparticles at pH 7.4 and 40 °C was reported to be 11.9 ± 5.1% in the sixth hour (Figure 7a) and 26.8 ± 3.4% in the 72nd hour (Figure 8A). This temperature was above the LCST and the hydrogel on the surface of the particles was in its collapsed state, which allowed the opening of the pores and the release of the drug from the inside. The hydrogel acted as a gatekeeper, and Dox was released only after the required conditions had been met. There was statistically significant difference in the amount of Dox released from MCM-41/AA-gPnVCL at the three investigated temperatures. The ANOVA showed that the release at 40 °C was significantly different from the release at 25 °C (*p* = 0.0060) and at 37 °C (*p* = 0.0061). These findings suggest that the thermosensitive release was maintained in the preparation of hybrid nanoparticles.

At pH 4.0 (Figure 7b and Figure 8B), all of the investigated nanocarriers exhibited increased release of Dox, which was expected due to its known better solubility in acidic media [54,55]. Such acidic conditions are expected for tumour cells because of their extensive metabolism [56]. The pristine MCM-41 loaded with Dox at pH = 4.0 showed a significant release with some burst effect in the first 6 h of dissolution (47.3 ±. 2.6% at 25 °C; 46.7 ± 2.0% at 37 °C; 48.3 ± 3.6% at 40 °C). There was no significant difference between the release at 40 °C and 25 °C (*p* = 0.81), as well as between the release at 40 °C and 37 °C (*p* = 0.79), and thus the unmodified nanoparticles possessed no temperature-sensitivity. Therefore, no actual sustained release was observed. In the acidic medium, the thermosensitive hydrogel AA-g-PnVCL loaded with Dox again completely released the API (96.8 ± 3.8%) within 6 h at 40 °C (Figure 7b). At temperatures lower than the LCST, a sustained release was observed, with lower amounts of Dox being released from the thermopolymer (25.3 ± 4.1% at 25 °C; 27.9 ± 3.5% at 37 °C). A comparison of the profiles between 25 °C and 40 °C as well as the ones between 37 °C and 40 °C showed significant differences (*p* < 0.05).

If we compare the release of Dox at 6 h at pH 4.0 from MCM-41, AA-g-PnVCL and MCM-41/AA-g-PnVCL (Figure 7b), sustained and temperature-triggered release from the hybrid nanoparticles was observed (29.9 ± 2.8%). The newly prepared hybrid nanoparticles under the simulated tumour conditions (pH = 4 and 40 °C) showed a sustained release without a burst effect in the initial phase (Figure 7b). Over time, a gradual controlled release was observed, and 88.9 ± 2.4% of the API was released within 72 h (Figure 8). This could lead to the assumption that no fluctuation in the concentration of Dox at the target site would be present. These findings support the expectations that combining two types of delivery systems, i.e., thermosensitive hydrogel and MSNs, will lead to superior properties and could improve the anticancer therapy.

On the basis of the data obtained, it can be assumed that the modification of MCM-41 particles with a thermopolymer allows a delayed and stimulus-triggered release compared with the hydrogel alone and unmodified particles. Providing very low drug release over time at a physiological pH of 7.4 at 37 °C (15.3 ± 6.3%) is a prerequisite for lowering the systemic toxicity of the novel MCM-41-AA-gPnVCL nanoparticles. Sustained and almost complete release (88.9 ± 2.4%) from the hybrid nanoparticles was observed only when an area with local tumour hyperaemia and an acidic pH was reached.

#### 2.2.9. Haemolysis Assay

**Figure 9:**
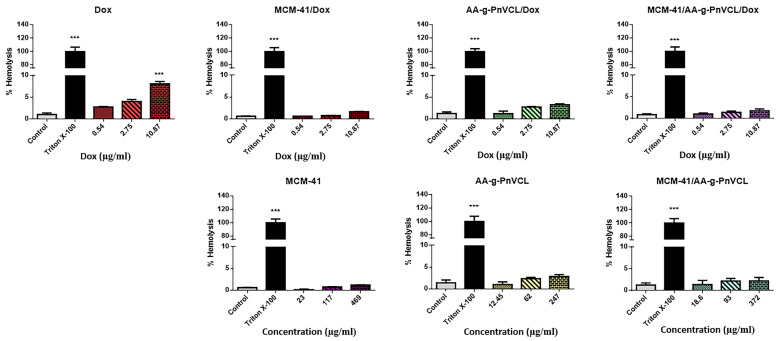
Haemolytic effects of free Dox (0.54–10.87 μM), Dox loaded in MCM-41, Dox loaded in AA-g-PnVCL, Dox loaded in MCM-41/AA-g-PnVCL (the concentrations of loaded Dox correspond to the concentrations of free Dox), and the free carriers MCM-41, AA-g-PnVCL and MCM-41/AA-g-PnVCL on human erythrocytes. The results are expressed as the mean ± SD of triplicate assays (n = 3). All groups were compared statistically vs. the untreated controls by one-way ANOVA with Dunnet’s post-test *** *p* < 0.001 vs. control.

A haemolytic assay is a widely used primary screening method for in vitro biocompatibility testing of new drug substances. The haemolytic activity of the tested compounds was compared with the effects of Triton X-100, as shown in Figure 9. Triton X-100 induced complete haemolysis (by 100% vs. the control) after 1 h of incubation. 

The experimental results showed the good hemocompatibility of all tested free carriers (MCM-41, AA-g-PnVCL and MCM-41/AA-g-PnVCL), because no haemolytic activity was observed, since the rate of haemolysis was lower than 5% (the accepted threshold level according to ISO 10993). Similarly, Dox (0.54, 2.75 and 10.87 µM) loaded in the nanoparticles (MCM-41-Dox, AA-g-PnVCL-Dox and MCM-41/AA-g-PnVCL-Dox) did not show a haemolytic effect, since the rate of haemolysis was lower than 5% (respectively, 1.6%, 3.3% and 1.8% vs. the untreated control). In contrast, the highest concentration of free Dox (10.87 µg/mL) was found to induce a slight increase in haemolysis by 8% vs. the untreated control.

In summary, empty nanoparticles and the Dox loaded in MCM-41, AA-g-PnVCL and MCM-41/AA-g-PnVCL did not show significant haemolytic effects. Our results are in accordance with the study of Saroj et al., who reported that pH-sensitive polyacrylic-acid-functionalized MCM-41 nanoparticles demonstrated hemocompatibility similar to that of the negative control group [57]. Zhao et al. reported the good biocompatibility of mesoporous silica nanoparticles MCM-41 with human erythrocytes compared with the highly haemolytic amorphous silica [58].

Thus, the results from our experiments confirmed the good hemocompatibility of the empty carriers, which is a promising perspective for parenteral application. Interestingly, we found that the loading of Dox in MCM-41, AA-g-PnVCL and MCM-41/AA-g-PnVCL nanoparticles improved its hemocompatibility compared with the effects of free Dox.

## 3. Conclusions

A hybrid, controlled drug delivery system based on AA grafted with PnVCL and attached to the surface of MCM-41 was obtained successfully. The newly synthesized hydrogel could efficiently serve as a gatekeeper of the MSNs, governing the drug’s release in response to the environmental temperature and pH. The model drug doxorubicin was almost completely released only under the simulated cancer tissue conditions (40 °C and pH = 4.0). In addition, the hydrogel on the surface of the silica particles formed internal cavities for drug molecules, which allowed a high loading efficiency (59%). The release profile was prolonged within a 72 h timeframe, which could limit the frequency of treatment. The haemolysis assay demonstrated the good biocompatibility of empty and Dox-loaded nanoparticles, since no significant haemolysis of red blood cells was observed.

All the results presented here suggest that the developed hydrogel-functionalized MCM-41/AA-g-PnVCL-based platform is an attractive candidate for stimulus-triggered and controlled drug release for chemotherapeutic agents. Once the preparation, successful “smart” release properties and safety upon parenteral application are proven, further studies would be necessary to evaluate its therapeutic efficacy in vitro and in vivo.

## 4. Materials and Methods

### 4.1. Materials

MCM-41, 3-aminopropyltriethoxysilane (APTES), succinic anhydride, anhydrous toluene, agar-agar powder and N-vinyl caprolactam (nVCL) were purchased from Sigma Aldrich Inc. (St. Louis, MO, USA). The 2,2′-azobis(2,4-dimethylvaleronitrile) (AMVN) and hydroquinone were purchased from TCI Europe (Zwijndrecht, Belgium). Ethanol, disodium hydrogen phosphate dihydrate and potassium dihydrogen phosphate were all purchased from Merck (Darmstadt, Germany). Deionized water was obtained by ion exchange.

### 4.2. Synthesis of Hydrogel-Functionalized MCM-41 Nanoparticles

#### 4.2.1. Synthesis of COOH-Modified MCM-41 Particles (MCM-41-COOH)

The carboxylation of MCM-41 was carried out in two steps. The first one included intermediate amination, in which 1 g of MSNs and 20 mL of 3-aminopropyltriethoxysilane (APTES) were mixed in ethanol for 5 h at 50 °C. Then a two-step washing process was conducted, first with ethanol and thereafter with deionized water. The resulting particles were dried at room temperature, followed by azeotropic dehydration. They were placed in toluene at 115 °C and the adsorbed water was removed.

The second step was the carboxylation of the amino-functionalized MCM-41. For this purpose, succinic anhydride (6.6 mmol) was added to the dispersion of the amino-modified MCM-41 in anhydrous toluene at 60 °C for 24 h. Carboxylation was performed by assuming that the MCM-41 was pre-functionalized with a 2 wt% amino content. The resulting modified particles were dried for 6 h by vacuum evaporation at 25 °C, and the obtained MCM-41-COOH particles were used for further thermopolymer attachment.

#### 4.2.2. Synthesis of MCM-41-COOH/AA-g-PnVCL Nanoparticles 

The formation of hydrogel containing AA and nVCL in a molar ratio of 20:1 by the free radical polymerization technique in the presence of AMVN as an initiator (AMVN: nVCL in a molar ratio of 1:20) is explained elsewhere [33]. To obtain the hybrid nanosystem, a modified direct polymerization method was applied according to the following procedure: AA (20 mg) was dissolved in 40 mL of distilled water at 90 °C with constant stirring. The MCM-41-COOH particles (250 mg) were homogeneously dispersed in the AA solution. Next, nVCL (140 mg) was dissolved in 40 mL of 95% ethanol and added to the mixture in a three-necked round bottomed flask in a nitrogen atmosphere for 60 min. Next, a solution of AMVN in 20 mL of 95% ethanol was added, and the nitrogen flow continued for another 15 min before the flask was closed. The reaction continued with ongoing stirring in a thermostatic paraffin bath (70 °C) for 16 h. Finally, the grafting procedure was terminated by adding a saturated solution of hydroquinone. The resulting MCM-41/AA-g-PnVCL particles were precipitated in acetone and separated by centrifugation (15,000 rpm). Removal of the homopolymer (PnVCL) was carried out by extraction with 95% ethanol for 24 h. Then the MCM-41/AA-g-PnVCL particles were dried under a vacuum to a constant weight and further subjected to characterization. A schematic representation of the procedure is shown in Figure 1.

### 4.3. Characterization of the Hydrogel, the MCM-41 Nanoparticles and the Hydrogel-Functionalized Nanoparticles (MCM-41/AA-g-PnVCL)

#### 4.3.1. Fourier Transform Infrared Spectroscopy (FT-IR)

A Thermo-Nicolet 400 FT-IR instrument equipped with an attenuated total reflectance (ATR) device (Thermo Fischer Scientific, USA was used to collect the IR spectra in the range of 4000–400 cm^−1^ with a resolution of 4 cm^−1^ for the free components as well as the grafted products.

#### 4.3.2. Transmission Electron Microscopy (TEM) and Scanning Electron Microscopy (SEM)

The size and structure of the gatekeeper-modified nanoparticles were characterized using transmission electron microscopy (JEOL JEM 2100 h STEM (200 kV; point resolution = 0.23 nm)). Samples were prepared by placing a water suspension of the nanoparticles on a polymer microgrid supported on a Cu grid. The water was further evaporated under a vacuum. The surface morphology was additionally examined on a scanning electron microscope (JSM 5510, JEOL, Tokyo, Japan) operating at 10 kV. Before imaging, the samples were coated with gold for 30 s using a sputter-coater (JSC 1200, JEOL, Tokyo, Japan) in an inert argon atmosphere.

#### 4.3.3. Thermogravimetric Analysis (TGA)

A PerkinElmer TGA4000 thermogravimeter was used to perform the TGA, and the operating conditions were as follows: argon gas at 60 mL/min, a temperature range from 40 °C to 820 °C and a heating rate of 10 °C/min. Specialized software (Pyris v.11.0.0.0449) was used for data collection and processing.

#### 4.3.4. X-ray Powder Diffraction (XRD)

XRD measurements were applied to characterize the nanoparticle crystallographic structure. The small-angle parts of the XRD patterns were collected from 0.3 to 8° 2θ using a knife-edge antiscatter screen attachment of the primary beam. Patterns were obtained on the Bruker D8 Advance diffractometer with Cu Kα radiation and a LynxEye detector (Bruker Corporation, Karlsruhe, Germany).

#### 4.3.5. Determination of the Lower Critical Solution Temperature (LCST) 

The characterization was performed via transmittance measurements on a UV spectrophotometer (Thermo Scientific Evolution 300, Madison, WI, USA) at 500 nm with a heating/cooling cycle step of 1 °C after incubating the samples for 5 min at each temperature. The light passed through the solution and the transmittance, as a percentage, was measured. At the LCST, a change in turbidity of the AA-g-PnVCL solution occurred.

#### 4.3.6. Dynamic Light Scattering (DLS)

The nanoparticles’ size, polydispersity index and zeta potential were determined using a Zetasizer (Zetasizer Nano ZS, Malvern Panalytical, Worcestershire, UK). The samples (0.1% *w*/*v*) were dispersed in distilled water, sonicated for 20 min and measured at a scattering angle of 90° and at 25 °C.

#### 4.3.7. Drug Loading and Loading Efficiency

The wet mixing method was used for loading the model drug Dox into the hybrid MCM-41/AA-PnVCL particles. The particles were mixed with Dox at a weight ratio of 1:1 in 10 mL of water on a magnetic stirrer for 2 h at 40 °C. Then they were allowed to cool to room temperature. After incubation, the samples were centrifuged at 14,000 rpm for 20 min, washed three times with ethanol and dried under a vacuum for 24 h. Quantitative loading of Dox was determined by analysing the obtained supernatant by UV-vis absorption at 480 nm and was calculated by the formula

(1) $$\mathrm{Drug}\;\mathrm{loading}\;\mathrm{efficiency}\;(\%)=\frac{A-B}{A}\times 100$$

where A is the total weight of Dox used for the drug-loading procedure and B is the weight of Dox which was present in the supernatant.

#### 4.3.8. In Vitro Drug Release Study

All samples including MCM-41/Dox, AA-g-PnVCL/Dox and MCM-41/AA-PnVCL/Dox in amounts equal to approximately 6 mg of Dox were placed in a dialysis bag (MWCO: 6–8 kDa, Spectra/Por^®^) with 3 mL of a phosphate buffer solution (PBS) (pH 4.0 and pH 7.4) and were immersed in 20 mL of a buffered medium with the corresponding pH. Drug release was studied using an incubator shaker at 25 °C, 37 °C and 40 °C. Aliquots were withdrawn, analysed by UV-vis spectroscopy at λ_max_ = 480 nm and replaced with fresh medium at predetermined time points. A control experiment was conducted with free Dox to confirm that the selected dialysis membrane ensured unrestricted diffusion of the released drug. The highest drug concentration in the dialysate was below 10% of the drug’s aqueous solubility, which is a prerequisite for “sink” conditions. The release study was performed in triplicate. The amount of Dox released was calculated on the basis of a previously obtained calibration curve in the corresponding PBS medium. One-way ANOVA was used to determine the significance of the differences in the amounts released. The significance level was set as *p* < 0.05.

#### 4.3.9. Haemolysis Assay

The haemolytic potential of the test substances was evaluated following the protocol described by Evans et al. [59]. Blood samples from healthy volunteers were obtained from a certified clinical laboratory. The experimental procedures were conducted according to the rules of the Institutional Ethics Committee (KENIMUS) of the Medical University of Sofia, Bulgaria [60]. The erythrocytes were separated from the blood by repeated centrifugation in a 0.9% NaCl buffer. Then the blood cells were resuspended in a phosphate buffer (pH 7.4). The test substances (at appropriate concentrations), 20% Triton X-100 (used as a positive control group) and the phosphate buffer (used as a negative control group) were pipetted into 96-well plates, and the erythrocyte suspension in a phosphate buffer was added to them. Then the plates were incubated for 1 h at 37 °C and centrifuged for 5 min at 500× *g*. The supernatant was moved to new 96-well plates, in which the absorbance of haemoglobin was measured at 430 nm in a Synergy 2 plate reader (BioTek Instruments, Inc., Highland Park, Winooski, VT, USA). The results obtained were presented as the percentage of haemolysis relative to the haemoglobin’s absorbance values in the positive controls; the haemoglobin absorbance of the negative controls was accepted as zero haemolysis. The substances that caused haemolysis below 5% (the acceptable haemolytic threshold is 5% according to ISO 10993-5) were considered to be biocompatible [61,62].

#### 4.3.10. Statistical analysis

Statistical analysis was performed by one-way ANOVA followed by Dunnett’s post hoc test. Statistical evaluation was performed using GraphPad 6 software. Differences were accepted to be significant at *p* < 0.001. All statistical analyses were carried out with Graph Pad 6 software. The results are expressed as the mean ± SD (n = 6) for 3 independent experiments.

## Figures and Tables

**Table 1:** Average size, polydispersity index (PDI) and zeta potential from the DLS analysis; mean ± SD, *n* = 3.

Parameter	MCM-41	MCM-41/AA-g-PnVCL	MCM-41/AA-g-PnVCL/Dox
Size, nm	217 ± 7.3	360 ± 8.4	369 ± 3.3
PDI	0.31	0.86	0.83
Zeta potential, mV	−37.1 ± 3.7	−26.6 ± 3.6	−19.8 ± 2.4

## Data Availability

Data are available from the authors (see the email of the corresponding author).
